# Alteration in sperm characteristics, endocrine balance and redox status in rats rendered diabetic by streptozotocin treatment: attenuating role of *Loranthus micranthus*

**DOI:** 10.1080/13510002.2018.1540675

**Published:** 2018-10-30

**Authors:** Azubuike P. Ebokaiwe, Omamuyovwi M. Ijomone, Sharon O. Osawe, Chukwuma J. Chukwu, Chukwunonso E.C.C. Ejike, Guolin Zhang, Fei Wang

**Affiliations:** a Department of Chemistry/Biochemistry and Molecular Biology, Federal University Ndufu-Alike Ikwo, Ikwo, Nigeria; b Department of Anatomy, School of Health and Health Technology, Federal University of Technology Akure, Akure, Nigeria; c Department of Biochemistry, Faculty of Applied Sciences, KolaDaisi University, Ibadan, Nigeria; d Department of Medical Biochemistry, College of Medicine, Federal University Ndufu-Alike Ikwo, Ikwo, Nigeria; e Key Laboratory of Natural Medicine and Clinical Translation, Chengdu Institute of Biology, Chinese Academy of Sciences, Chengdu, People's Republic of China

**Keywords:** *Loranthus micranthus*, spermiogram, diabetes, oxidative stress, apoptosis, steroid hormones, male rats

## Abstract

**Objectives:***Loranthus micranthus* is widely used in Nigerian folklore treatment of male infertility and diabetes complications. We investigated this claim in rats rendered diabetic by streptozotocin (STZ).

**Methods:** Induction of diabetes mellitus in adult male Wistar rats was by intraperitoneal injection of STZ (60 mg/kg). The diabetic rats were thereafter treated orally once/day with 5 mg/kg Gilbenclamide or *L. micranthus* (100 mg/kg or 200 mg/kg) and monitored for 14 days. Clinical observations, hormonal profile, oxidative stress parameters, glucose metabolism enzymes, histopathological examination, apoptotic marker immunoreactivity and western blotting in testes and sperm parameters were evaluated to examine effects of *L. micranthus* on STZ-diabetic rats.

**Results:***L. micranthus* treatment significantly reduced the blood glucose level (45.9% and 84.7% on the 7th and 14th post-treatment days, respectively); increased antioxidant status, improved microarchitecture of testes, reduced lipid peroxidation and increased BCl-2 protein expression in diabetic rats relative to control. Furthermore, treatment with *L. micranthus* increased steroidogenic enzymes activities, levels of steroid hormones and improved sperm quality, relative to control.

**Conclusion:** The anti-diabetic and aphrodisiac properties exhibited by *L. micranthus* could be contingent on its ability to restore a balance to the compromised redox status that characterizes male reproductive dysfunction in diabetes.

## Introduction

The idea of turning locally grown plants into medicines for the local population has been a widely acceptable practice, over the years. Indeed, traditional and western medicine is rich in examples where biologically active plants or plant parts are simply ‘mashed’ and applied topically, orally etc. for treatment of various ailments. Hence, to understand potential ways to recover the quality of life for diabetic patients and reversing diabetic complications necessitate investigations into the nature’s treasure of green gold (herbs) [1,2]. Diabetes mellitus is among the severe chronic diseases globally [3]. Several studies on the hypoglycemic activity of the aqueous methanolic extracts of *L. micranthus* leaves were found to possess significant dose-dependent activities [1,4,5]. *L. micranthus* is believed to be an ‘all purpose’ herb due to its rich traditional uses and it has been widely used in ethnomedicine for treating/managing various ailments, ranging from hypertention, cancer, to treatment of male infertility [6].

Several reports have shown that *L. micranthus* is rich in various classes of flavonoids [7–9]. Interestingly, there is mounting evidence indicating that flavonoids from plant origin can augment glucose metabolism, lipid profile and regulate hormones and enzymes in human body, further protecting humans from diseases like obesity, diabetes and their complications [10]. One of the mechanisms to support this claim is that flavonoids have the ability to scavenge free radicals [11]. Of particular interest is the apparent relation between diabetes and oxidative stress/inflammation [12,13] and the potential for flavonoids to protect the body against free radicals and other pro-oxidative compounds [14,15]. It is therefore biologically justifiable to evaluate the protective role of *L. micranthus* on complications of diabetes along the testicular axis as studies to this effect are lacking. This will help to decipher the pharmacological potentials and encourage applications and consumption of flavonoid-rich plant products such as *L. micranthus* which could lessen the risk of diabetes complications as it pertains to male infertility [16,17].

Male reproductive function is a targeted physiological process mostly damaged by diabetes [18] due to the high susceptibility of testicular microenvironment to oxidative stress. The commonest of non-lethal complications in diabetic men is infertility. Studies have shown that several pathological and biochemical alterations accompanying diabetes could lead to deficits in male fertility [19]. Diabetes has been reported to induce metabolic alterations, disrupting the endocrine system, with a subsequent dysfunction of the hypothalamus–pituitary testicular (HPT) axis [20] that may lead to impairment of the male reproductive health [21–23]. The Sertoli cells (SCs) are known to produce metabolic precursors essential for germ cells development [24] and the process is exceedingly reliant on glucose uptake and lactate production [25]. From the biochemical point of view the metabolism of carbohydrates, specially glucose, is crucial for male reproductive health, so the preservation of testicular glucose metabolism redox is of particular relevance; for continuity of spermatogenesis [26,27]. Hence, there is need to develop therapeutic candidates with potentials to maintain testicular glucose metabolism since alterations in these mechanisms may herald male infertility [25–29].

Most attention in the pharmaceutical industries these days is on plant products, due to their low or apparently no side effects. Phytochemicals are non established nutrients with biologically active components, found in plants which confer significant protection against degenerative diseases [1]. Bioactive plant compounds are known to prevent diseases by inhibiting cellular damage induced by oxidative stress [31]. More so, phytochemicals have been found to be useful in pharmaceutical industries where they are harnessed in drug development which begins with identification of the active mechanisms, elaborate biological assays and dosage formulations followed by clinical studies to establish the safe dose and pharmacokinetic profile of the drug [2].

The present study was designed to further understand the mechanism involved in the ameliorative effect of *L. micranthus* along the testicular axis in diabetes complication and to the best of our knowledge there is no existing report on this. The acclaimed health beneficial effects of *L. micranthus* leaves on diabetes in folklore medicine, prompted the present mechanistic investigation into its effect on hyperglycemia mediated testicular dysfunction in rats rendered diabetic by streptozotocin treatment.

## Materials and methods

### Drug and chemicals

STZ, thiobarbituric acid (TBA), Bcl-2 antibody, 1-chloro-2,4-dinitrobenzene (CDNB), epinephrine, reduced glutathione (GSH), 5,5-dithiobis-2-nitrobenzoic acid (DTNB), NADPH, NADP and hydrogen peroxide were purchased from Sigma Chemical Co. (St Louis, MO, U.S.A.). All other reagents were of analytical grade and were obtained from the British Drug Houses (Poole, Dorset, UK).

### Plant collection and identification

Harvest of *L. micranthus* leaves were carried out in July 2016, from a forest around AE-FUNAI, Ikwo, Nigeria. The authentication of leaves as *L. micranthus* was done at the Department of Botany, University of Ibadan, Ibadan, where a voucher specimen already exists in the herbarium.

#### Preparation of *L. micranthus* aqueous-methanolic extract

Fresh *L. micranthus* leaves were air dried and crushed to powder in an electric grinder. Afterwards 100 g of the powder was suspended in 300 ml of aqueous-methanol (1:2) and kept in an incubator at 25°C for 36 h. The slurry was stirred intermittently for 2 h and left overnight. The mixture was then filtered, the filtrate obtained was dried by low pressure and 9 g of residue was collected. This was suspended in water in a fixed dose and used for treatment.100 mg/ml stock solution of *L. micranthus* leaves were prepared fresh every other day with distilled water during this study.

### Experimental animals

Forty adult male Wistar rats (6–8 weeks old, 150 ± 6 g) obtained from the Animal house, Federal University Ndufu-Alike Ikwo, Nigeria and Chengdu dachuo biotechnology Co., Ltd, China were used for this study. The rats were housed in plastic cages placed in a well-ventilated rat house, provided rat chow and water *ad libitum* and subjected to the natural photoperiod of 12 h light/dark. All the animals received humane care according to the criteria outlined in the Guide for the Care and Use of Laboratory Animals’ prepared by the National Academy of Science (NAS) and published by the National Institute of Health . The experiment was performed in accordance with the US NAS guidelines and approval of Institutional Animal Ethics Committee of Federal University Ndufu-Alike Ikwo.

### Experimental induction of diabetes

Rats were rendered diabetic by a single intraperitoneal administration of STZ (60 mg/kg) in freshly prepared citrate buffer (0.1 M, pH 4.5) after an overnight fast. The rats were provided food and water *ad libitum* after STZ administration. Following 72 h of STZ injection, blood samples were collected through the tail vein and blood glucose levels were determined with an Accu-check glucometer (Roche Diagnostics GmbH, Mannheim, Germany). The rats with fasting blood glucose levels above 230 mg/dl were considered as diabetic and selected for the study.

### Experimental procedure

Animals were randomly allocated into five groups of eight rats each and administered the following:

Group I: control (non diabetic) rats received citrate buffer at 2 ml/kg BW. Group II: diabetic rats received citrate buffer at 2 ml/kg BW. Group III: diabetic rats were treated with glibenclamide at 5 mg/kg BW. Group IV: diabetic rats were treated with *L. micranthus* leaves at 100 mg/kg BW. Group V: diabetic rats treated with *L. micranthus* leaves at 200 mg/kg BW. All administrations were done orally for 14 consecutive days.

Fasting blood glucose levels and body weights were monitored during the treatment period. The dose of *L. micranthus* leaves was selected from the earlier data published elsewhere [5]. Twenty-four hours following the last treatment, the overnight-fasted rats were sacrificed by cervical dislocation, and blood was collected from the retro-orbital venous plexus. Serum samples were separated from blood cells by centrifugation at 3000 g for 10 min and hormonal concentrations determined using Robonic S 2000 ELISA strip reader (Southampton, UK). The left testes were removed, weighed and stored at –20°C for further biochemical estimations. The right testes were fixed in Bouin’s solution for subsequent histological processing.

### Determination of sperm parameters

The progressive motility of the sperm from the rats was measured within 2–4 min after sacrifice following the method of Zemjanis [30]. Epididymal sperm number (ESN) was obtained by mincing the caudal epididymis in distilled water and filtering through a nylon mesh. The sperm were counted by a hemocytometer using the improved Neubauer (Deep 1/10 m; LABART, Munich, Germany) chamber according to Pant and Srivastava [31]. A portion of the sperm suspension placed on a glass slide was smeared out with another slide and stained with Wells and Awa’s stain (0.2 g eosin along with 0.6 g fast green were dissolved in distilled water and ethanol in a 2:1 ratio) for morphological examination. The sperm viability was determined using a stain containing 1% eosin and 5% nigrosines in 3% sodium citrate dehydrate solution.

### Estimation of testicular antioxidant system

The testes of the rats were homogenized in buffer (50 mM Tris–HCl) pH 7.4, containing 1.15% potassium chloride, and the homogenate was centrifuged at 10,000 g for 15 min at 4°C. The supernatant was thereafter collected for the determination of protein concentration by the method of Lowry et al. [32]. Superoxide dismutase (SOD) activity was determined according to the method described by Misra and Fridovich [33] and catalase (CAT) activity was estimated using hydrogen peroxide as a substrate according to the method of Clairborne [34]. Reduced glutathione (GSH) was estimated at 412 nm according to the method described by Jollow et al. [35]. GSH-Px was determined by the method of Rotruck et al. [36]. Lipid peroxidation was quantified as malondialdehyde (MDA) according to the method described by Farombi et al. [37].

### Estimation of plasma hormones concentration and steroidogenic enzyme assays

Plasma levels of Testosterone, FSH and LH were determined using BIO-RAD immunoassay kits (Alexandre, London, UK) according to the manufacturer’s instructions. The sensitivity of the testosterone assay was 0.07 ng/ml and with negligible cross reactivity with other androgen derivatives like androstenedione, 5a-dihydrotestosterone, and methyl testosterone. The intra-assay coefficients of variation for the testosterone assay were 4.1%. The sensitivity of LH was 0.06 ng at 80% whereas the FSH sensitivity was 0.05 ng at 98%. The intra-assay coefficients of variation were 3.1% for LH and 3.7% for FSH. The activity of 3β-HSD and 17β-HSD were measured by the method of Bergmeyer [38].

### Evaluation of testicular glucose metabolism enzymes activities

Testicular hexokinase activity (cytoplasmic fraction) was determined according to the method described by Branstrup et al. [39]. Glucose-6-phosphate dehydrogenase activity was estimated by the method of Dawson et al. [40]. LDH activity was determined using a commercial assay kit (Promega, Madison, U.S.A.) and following the manufacturer’s instructions.

#### Histology and histomorphometry

Testis tissues fixed in 10% neutral buffered formalin were processed for routine histological preparations. Sections were obtained from paraffin blocks and stained with haematoxylin and Eosin (H&E). Stained sections were observed under a digital light microscope (OMAX 3MP Digital Compound Microscope), at various magnifications and photomicrographs taken. Histological changes to the seminiferous tubules and adjacent interstitial areas were observed (Figure 9-I). For histomorphometry, histopathological changes in 50 seminiferous tubules for each section per animal were examined under a light microscope at ×400 and scored as follows; +3 for intact seminiferous tubules with sperm cells in the lumen and intact generations of spermatogenic cells, +2 for seminiferous tubules with reduced or no sperm cells in the lumen, and mild loss of generations of spermatogenic cells, +1 for seminiferous tubules with complete or severe loss generations of spermatogenic cells, and sertoli cells. Average score for each animal were calculated (Figure 9-II). Additionally, diameter of the more circular seminiferous tubules (Figure 9-II) and cell count of Leydig cells in adjacent interstitial areas were determined using image analysis software (Image J, NIH, U.S.A.).

#### Bcl-2 immunohistochemistry

Formalin-fixed and paraffin-embedded tissues were sectioned at 4 µm for immunohistochemistry. Immunohistochemical procedures were performed using LSAB^®^ HRP kit (Dako, Ltd) and anti-mouse Bcl-2 monoclonal antibody (Dako, Ltd). In brief; following deparaffinization and rehydration of sections, antigen retrieval was performed using preheated buffer solution of citrate (pH 6.0) and EDTA (pH 9.0) for 10 min, followed by protein block using 3% Hydrogen peroxide for 15 min. Section was incubated in primary antibody (anti-mouse Bcl-2) in a humidified chamber at room temperature for 1 h. Sections were then incubated at room temperature in *biotinylated link* for 15 min, and then in streptavidin-HRP for 15 min. Section where incubated for 5 min in diaminobenzidine substrate solution for chromogen reaction, and counterstained in Gill’s haematoxylin for 10 s. For quantification of Bcl-2 immunoreactivity, digital photomicrographs where imported unto the Image J software, and percentage of positive (DAB) stained nuclear area was quantified using the ImmunoRatio plugin. The ImmunoRatio application, computes the percentage of positively stained nuclear area by means of a colour deconvolution algorithm that separates the staining component (in this instance; the DAB chromogen and the haematoxylin counterstaining) and adaptive thresh holding for nuclear area segmentation [41–43].

#### Bcl-2 western blot assay

Testes lysates were prepared in the lysis buffer (50 mm Tris, pH 7.4 containing 0.15 M NaCl,10% glycerol (v/v), 1% NP-40 (v/v), 1 mm sodium fluoride, 1 mm sodium orthovanadate, 1 mm PMSF, 1 mm EDTA, 150 mm bestatin, 1 mm leupeptin and 1 mm aprotinin) using a tissue: buffer ratio of 1:5. After homogenization, samples were first centrifuged at 1000 × g for 2 min to remove large tissue fragments and then centrifuged again at 10,000 × g for 15 min. The supernatants were collected and stored at –80°C. The protein concentration was determined by Lowry’s method [32] and equal quantities of protein were loaded per lane and subjected to 10% sodium dodecyl sulfatepolyacrylamide gel electrophoresis [Mini Protean II System, Bio-Rad, Berkeley, CA, U.S.A.] as described by Laemmli [44]. Electrophoresis was performed at 80–120 V and the resolved proteins were electrophoretically transferred onto a nitrocellulose membrane (NYTRAN, Keene, NH, U.S.A.) in the transfer buffer (0.2 mol/l glycine, 25 mm Tris and 20% methanol). The membranes were incubated in a blocking buffer (phosphate-buffered saline (PBST) containing 0.1% (v/v) Tween-20 and 5% (w/v) non-fat dry milk powder) for 1 h at room temperature, followed by incubation in Bcl-2 primary antibodies (SAB, Ltd). Anti-Bcl-2 was diluted at 1/100, The incubation with the primary antibodies was carried out overnight at 4°C. The following day, blots were washed in PBST and incubated for 1.5 h at room temperature with horseradish peroxidase (HRP)-conjugated anti-rabbit IgG (Proteintech, Ltd) (1:3000 dilution). Immunodetection of proteins was revealed by ImageQuant LAS 500 (Japan), and resulting immunospecific bands were quantified by densitometry.

### Statistical analysis

Data were expressed as mean ± SEM. Data comparisons were performed using One-way ANOVA, followed by Student Newman–Keuls (SNK) for post hoc. GraphPad Prism (Version 5.03, GraphPad Software, U.S.A.) was used for charts and analysis. Statistical significance was set at *p *< 0.05.

## Results

### Effects of *L. micranthus* on body, organ weights and blood glucose levels

**Figure 1.**
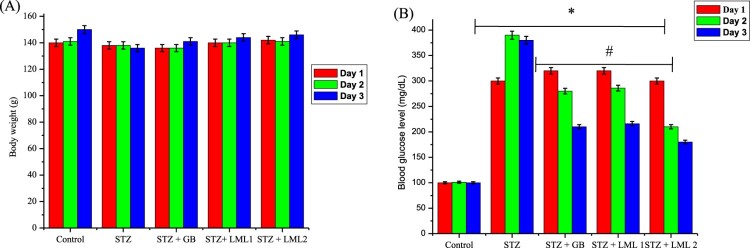
Effects of *Loranthus micranthus* on body weight (A) and blood glucose levels (B) in control and STZ-induced diabetic rats. Each bar represents mean ± SEM of eight rats. *p < 0.05 compared to control. #*p* < 0.05 compared to diabetic control group. STZ, 60 mg streptozotocin; STZ + GB, 60 mg STZ + 5 mg Gilbenclamide; STZ + 100 mg, 60 mg STZ + 100 mg LM extract; STZ + 200 mg, 60 mg STZ + 200 mg LM extract.

The body and organ weights of the treated groups at various days (1, 7 and 14th day of the study) are represented in Figure 1(A) and Table 1. There was a consecutive decrease in body weight of rats rendered diabetic by streptozotocin treatment from day 7 (0.87%) and continued at day 14 (3.66%). Oral administration of 5 mg GB also showed a weight loss of about 0.89% at day 7 but recorded weight gain of about 0.83% at day 14. However oral administration of 100 and 200 mg/kg body weight of *L. micranthus* showed a weight gain of (0.77% and 0.98%) and (2.44% and 5.12%) at various days, respectively. The data on the organ weights, presented in Table 1, showed that there was a significant (*p* < 0.05) decrease in the weights of testes for diabetic untreated group. However, treatment with GB and *L. micranthus* improved organ weight relative to control.

**Table 1.** Effect of *L. micranthus* (LM) extract on testes weight in STZ-induced diabetic rats.

	Control	STZ	STZ + GB	STZ + 100 mg/kg LM	STZ + 200 mg/kg LM
Testes (g)	1.43 ± 0.01	0.78 ± 0.02*	1.07 ± 0.00#	1.01 ± 0.01#	1.44 ± 0.01#

The data are expressed as mean ± SEM for eight rats per group after 14 days treatment period. *p < 0.05 against control. #*p* < 0.05 against diabetic control (STZ).

The blood glucose level of the control and experimental rats at day 1 and on the 7th and 14th days of the study is presented in Figure 1(B). The STZ-treated diabetic rats showed hyperglycemia as confirmed by a significant (*p* < 0.05) increase in the blood glucose level when compared with the control rats. The increase in the blood glucose level of STZ-induced diabetic rats was 200% to 260% above the control rats from day 3 to 14th day. However, oral administration of GB reduced the blood sugar levels from 180% to 94% compared to the control group, whereas *L. micranthus* extract at 100 mg/kg and 200 mg/kg resulted in a blood glucose lowering activity from 199% to 100% and 180% to 54% compared with control and diabetic group on the 7th and 14th post-treatment days, respectively.

### Effects of *L. micranthus* on testicular antioxidant system

**Figure 2.**
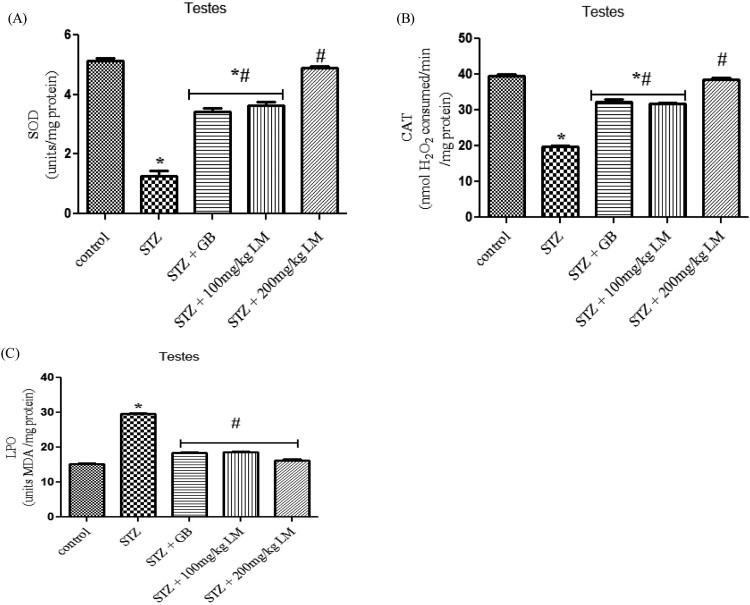
Effects of *L. micranthus* on activities of SOD (A), CAT (B) and LPO (C) in control and STZ-induced diabetic rats. Each bar represents mean ± SEM of eight rats. *p < 0.05 compared to control. #*p* < 0.05 compared to diabetic control group. STZ, 60 mg streptozotocin; STZ + GB, 60 mg STZ + 5 mg Glibenclamide; STZ + 100 mg, 60 mg STZ + 100 mg LM extract; STZ + 200 mg, 60 mg STZ + 200 mg LM extract.

**Figure 3.**
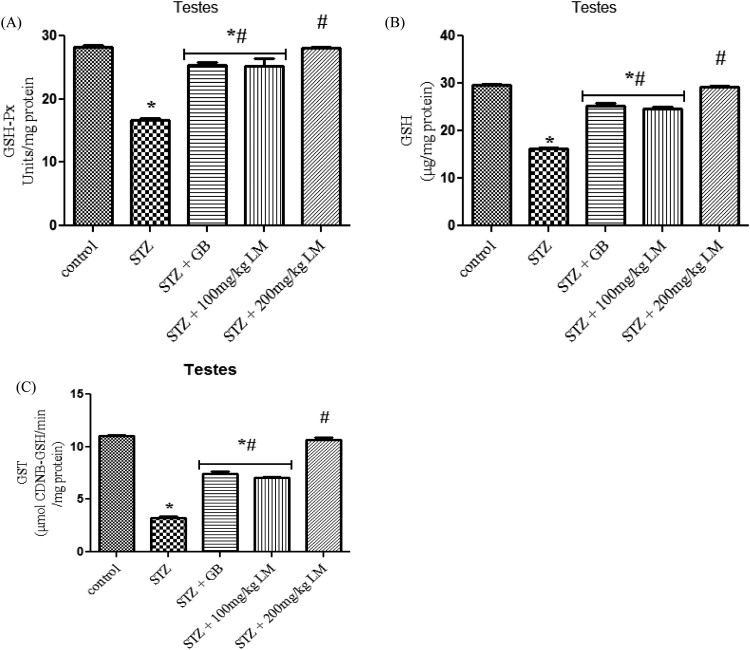
Effects of *L. micranthus* on activities of GSH-Px (A), GSH (B) and GST (C) in control and STZ-induced diabetic rats. Each bar represents mean ± SEM of eight rats. *p < 0.05 compared to control. #*p* < 0.05 compared to diabetic control group. STZ, 60mg streptozotocin; STZ + GB, 60 mg STZ + 5 mg Glibenclamide; STZ + 100 mg, 60 mg STZ + 100 mg LM extract; STZ + 200 mg, 60 mg STZ + 200 mg LM extract.

A significant (*p* < 0.05) decrease in activities of SOD, CAT and levels of MDA were observed in testes, of rats rendered diabetic by STZ treatment. Oral administration of *L. micranthus* extract ameliorated the decrease in activities of the enzymes and levels of MDA mostly at 200 mg/kg of the extract, whereas treatment with GB and 100 mg/kg *L. micranthus* showed significant (*p* < 0.05) improvement at day 14 when compared with diabetes group (Figure 2(A–C)). The levels of GSH (Figure 3(B)) and activities of glutathione antioxidants in Figure 3(A,C) (GSH-Px and GST) decreased significantly (*p* < 0.05) in diabetes group when compared with control. Moreover, a significant recovery was recorded in GB and 100 mg/kg *L. micranthus* treated group when compared with diabetes group while 200 mg/kg *L. micranthus* recovered most when compared with control.

### Effects of *L. micranthus* on activities of glucose metabolism enzymes; hexokinase, G-6-PD and LDH

**Figure 4.**
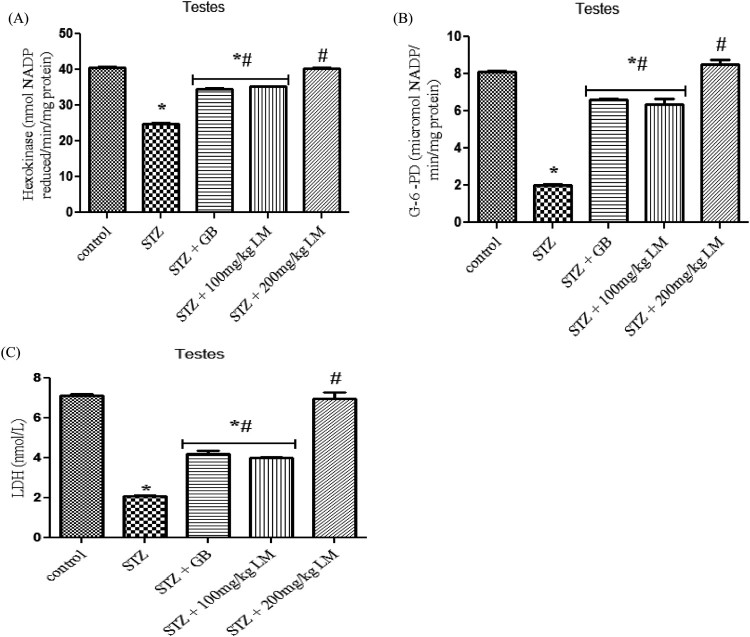
Effects of *L. micranthus* on activities of glucose metabolism enzymes Hexokinase (A), G-6-PD (B) and LDH (C) in control and STZ-induced diabetic rats. Each bar represents mean ± SEM of eight rats. *p < 0.05 compared to control. #*p* < 0.05 compared to diabetic control group. STZ, 60 mg streptozotocin; STZ + GB, 60 mg STZ + 5 mg Glibenclamide; STZ + 100 mg, 60 mg STZ + 100 mg LM extract; STZ + 200 mg, 60 mg STZ + 200 mg LM extract.

The activities of glycolytic enzymes (hexokinase, glucose-6-phosphate dehydrogenase, and lactate dehydrogenase) in the testes of control and experimental rats are shown in Figure 4(A–C), respectively. The activity of G-6-PD, hexokinase and LDH were observed to be significantly decreased, in diabetic rats, when compared with control rats (*p* < 0.05). Oral administration of GB (5 mg/kg body weight) and *L. micranthus* (100 mg/kg body weight) for 14 days significantly reversed these values to near normal for GB and 100 mg/kg *L. micranthus*. However, a total recovery was seen in 200 mg/kg *L. micranthus* group compared with control.

### Effects of *L. micranthus* on levels of endocrine hormones and steroidogenic enzymes activity

**Figure 5.**
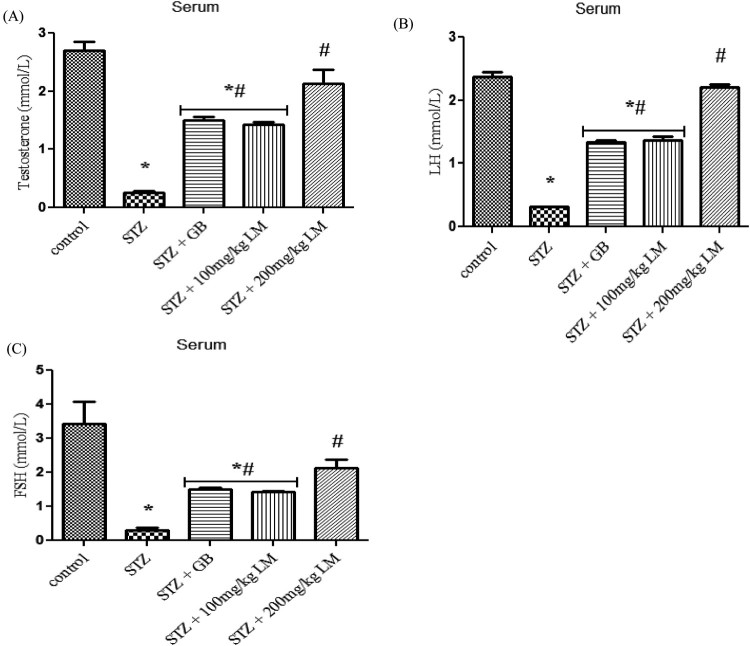
Effects of *L. micranthus* on levels of endocrine hormones in control and STZ-induced diabetic rats. Each bar represents mean ± SEM of eight rats. *p < 0.05 compared to control. #*p* < 0.05 compared to diabetic control group. STZ, 60 mg streptozotocin; STZ + GB, 60 mg STZ + 5 mg Glibenclamide; STZ + 100 mg, 60 mg STZ + 100 mg LM extract; STZ + 200 mg, 60 mg STZ + 200 mg LM extract.

**Figure 6.**
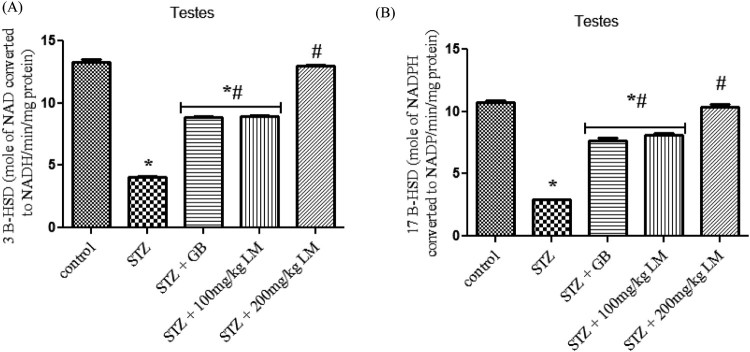
Effects of *L. micranthus* on activities of steroidogenic enzymes in control and STZ-induced diabetic rats. Each bar represents mean ± SEM of eight rats. *p < 0.05 compared to control. #*p* < 0.05 compared to diabetic control group. STZ, 60 mg streptozotocin; STZ + GB, 60 mg STZ + 5 mg Glibenclamide; STZ + 100 mg, 60 mg STZ + 100 mg LM extract; STZ + 200 mg, 60 mg STZ + 200 mg LM extract.

The influence of *L. micranthus* extracts on serum concentrations of testosterone, LH, and FSH in rats rendered diabetic by STZ treatment are presented in Figure 5(A–C), respectively. Circulatory concentrations of the hormones in rats rendered diabetic by STZ treatment were significantly decreased when compared with those of control rats. However, oral treatment with *L. micranthus* extract and GB at 100 mg/kg and 5 mg/kg significantly (*p* < 0.05) improved the suppression of these hormones by elevating their levels to near control in spite of diabetic state. Moreover at 200 mg/kg *L. micranthus* administration, a better recovery was observed when compared with other treated groups and control group. The activities of enzymes involved in steroidogenesis (3-β and 17-βhydroxysteroid dehydrogenases) in the testes of control and experimental rats are shown in Figure 6(A,B). The activity of the enzymes was observed to be significantly decreased, in diabetic rats, when compared with control rats (*p* < 0.05). Oral administration of GB (5 mg/kg body weight) and *L. micranthus* (100 mg/kg body weight) for 14 days significantly reversed these values to near normal. However no significant (*p* < 0.05) difference was seen in 200 mg/kg *L. micranthus* group compared with control.

### Effects of *L. micranthus* on sperm parameters

**Figure 7.**
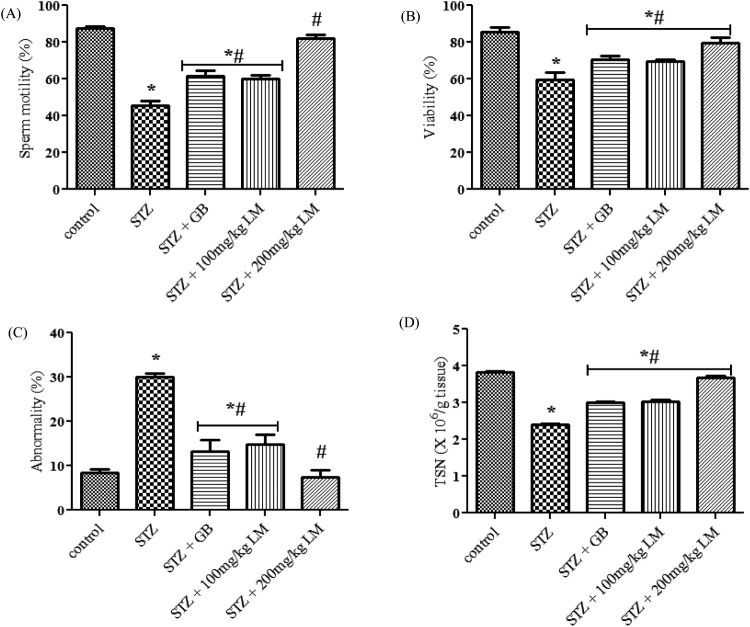
Effects of *L. micranthus* on sperm parameters in control and STZ-induced diabetic rats. Each bar represents mean ± SEM of eight rats. *p < 0.05 compared to control. #*p* < 0.05 compared to diabetic control group. STZ, 60 mg streptozotocin; STZ + GB, 60 mg STZ + 5 mg Glibenclamide; STZ + 100 mg, 60 mg STZ + 100 mg LM extract; STZ + 200 mg, 60 mg STZ + 200 mg LM extract.

The spermiogram of the experimental rats are represented in Figure 7(A–D). The percentage of sperm progressive motility, viability, abnormality and TSN decreased significantly in rats rendered diabetic by streptozotocin treatment (*p* < 0.05). The percentage of abnormal sperm with morphological defects increased significantly in rats rendered diabetic by streptozotocin treatment when compared with the control rats. The major abnormalities in rats rendered diabetic by streptozotocin treatment group include tailless heads, curved mid-pieces, and bent mid-pieces. However, the sperm progressive motility and viability were restored to control levels following treatment with 200 mg/kg body weight of *L. micranthus* when compared with the rats rendered diabetic by streptozotocin treatment. The decrease in the sperm viability and TSN in rats rendered diabetic by streptozotocin treatment were not completely restored when compared with the control rats within the study period. Administration of *L. micranthus* and Glibenclamide showed a noticeable augmentation/restoration of spermatogenesis and steroidogenesis.

### Effects of *L. micranthus* on histology and histomorphometry

**Figure 8.**
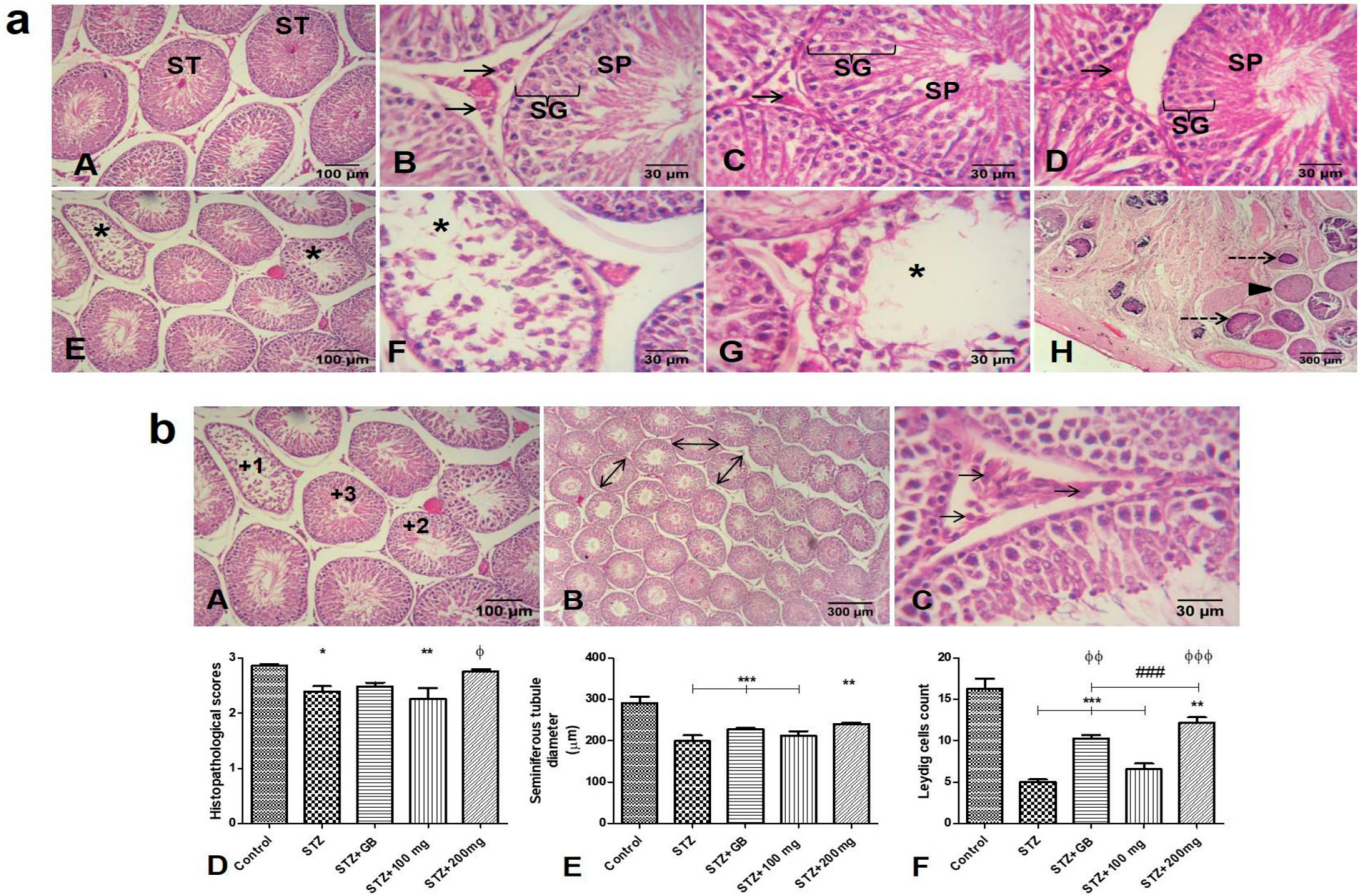
(a) Histological analysis (H & E). Mostly intact seminiferous tubules (ST) are observed in control (A & B), STZ-diabetic rats treated with glibenclamide (C) or 200 mg/kg extract (D). Observe intact spermatogenic generations of cells (SG), spermatozoa (SP) in lumen of tubules, and Leydig cells (arrows) in the interstitial supporting tissues between tubules. Degeneration and loss of tubular constituents (asterisks) is observed in tubules of STZ-diabetic untreated rats (E & F), and diabetic rats treated with 100 mg/kg extract (G). Particularly, an animal in untreated diabetic group presented extensive necrosis of the testis, with several tubules totally necrosed (arrowhead). Additionally, some necrosed tubules showed calcification within (dashed arrows) (H). (b) *Top* – representation of histomorphometry: (A) Histopathological scores for testicular damage; +3 for intact seminiferous tubules with sperm cells in the lumen and intact generations of spermatogenic cells, +2 for seminiferous tubules with reduced or no sperm cells in the lumen, and mild loss of generations of spermatogenic cells, +1 for seminiferous tubules with complete or severe loss generations of spermatogenic cells, and sertoli cells. (B) Measurement of seminiferous tubules diameter (double arrows). (C) Interstitial cells of Leydig (arrows). *Bottom* – Histomorphometric analysis of histopathology (D), seminiferous tubule diameter (E), and count of Leydig cells (F). *p < 0.05, ** *p* < 0.01, *** *p* < 0.001 compared to control; ### *p* < 0.01 compared to untreated STZ-diabetic; ϕϕ *p* < 0.01, ϕϕϕ *p* < 0.001 compared to STZ-diabetic treated with 100 mg/kg extract.

Figure 8(a): Histology; Control rats showed mostly intact seminiferous tubules, with intact spermatogenic generations of cells and spermatozoa in lumen of tubules. Additionally, the Leydig cells with their characteristically polygonal or rounded shape, rounded nuclei and dispersed chromatin are obvious in the adjacent interstitial tissues (A and B). Diabetic rats treated with glibenclamide and 200 mg/kg extract showed improved histology of the testis, with many intact tubules, and spermatozoa filled lumen (C and D). Untreated STZ-diabetic rats presented many seminiferous tubules with degeneration and loss of spermatogenic cells as well as loss of luminal spermatozoa. Additionally, there was loss or atrophy of adjacent interstitial Leydig cells. Particularly, extensive necrosis of the testis is shown in one animal (E and F). Calcification was observed in several necrosed seminiferous tubules (H). On the other hand, treatment with 100 mg/kg extract did not improve microstructural anomalies observed in diabetic rats (G). Figure 8(b): Results of histomorphometric analysis showed that there were significant histopathologic alterations in untreated STZ-diabetic rats (*p* < 0.05) and 100 mg/kg extract treated diabetic rats (*p* < 0.01) compared to control. However, treatment with glibenclamide and 200 mg/kg extract attenuated these histopathologic alterations as these groups showed no significant alterations compared to control (D). Relative to control, diameter of seminiferous tubules were significantly reduced in untreated STZ-diabetic rats, glibenclamide and 100 mg/kg extract treated diabetic rats (*p* < 0.001), as well as 200 mg/kg extract treated diabetic rats (*p* < 0.01) (E). Number of Leydig cells was significantly reduced in untreated STZ-diabetic rats, however, treatment with glibenclamide and 200 mg/kg extract significantly improved number of Leydig cells when compared to both untreated STZ-diabetic rats and 100 mg/kg extract treated diabetic rats (F).

### Effects of L. micranthus on Bcl-2 immunoreactivity in testes

**Figure 9.**
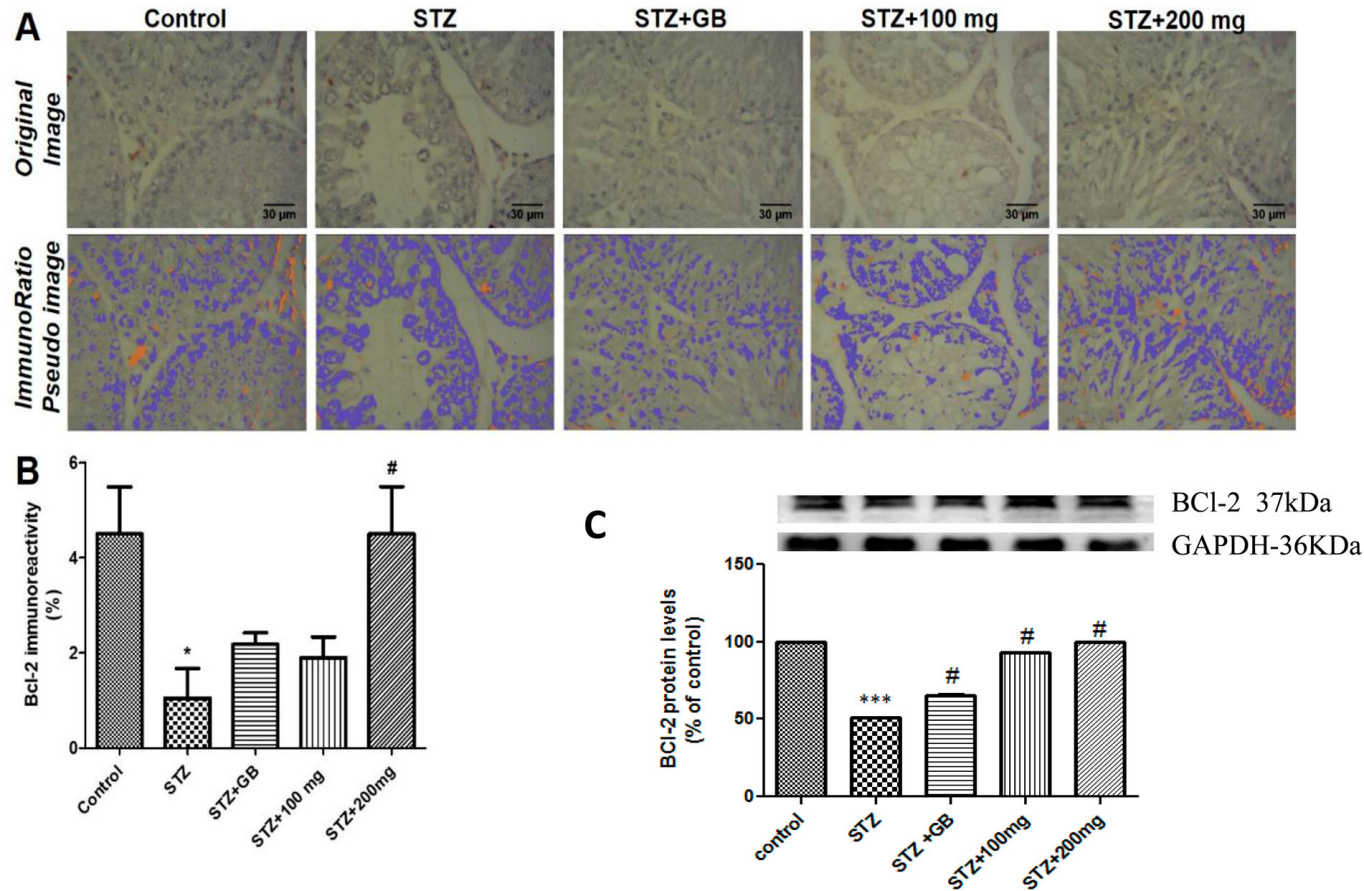
(A) Immunoreactivity of Bcl-2 in testis of control and treated rats. Top panel show original photomicrographs, while bottom panel show ImmunoRatio Pseudo-coloured images to quantify percentage of positive Bcl-2 immunoreactivity. (B) Analysis of ImmunoRatio quantification of Bcl-2 immunoreactivity. (C) Western blot evaluation of Bcl-2 protein levels. *P < 0.05 compared to control; ***p < 0.001; #*p* < 0.01 compared to untreated STZ-diabetic.

There was a significant decrease (*p* < 0.05) of Bcl-2 immunoreactivity in testes of untreated rats rendered diabetic by STZ treatment compared to control. Treatment with 200 mg/kg of the extract significantly increased Bcl-2 immunoreactivity (*p* < 0.05) in STZ-induced diabetic rats. However, treatment with glibenclamide and 100 mg/kg extract showed no significant effect on Bcl-2 immunoreactivity in diabetic rats (Figure 9(A,B)).

### Effects of *L. micranthus* on Bcl-2 protein levels in testes

There was significant decrease (*p* < 0.05) of Bcl-2 protein levels in testes of untreated rats rendered diabetic by STZ treatment compared to control. Treatment with glibenclamide, 100 and 200 mg/kg extract showed a significant increase (*p* < 0.05) in the protein levels when compared with the untreated group (Figure 9(C)).

## Discussion

Diabetes, a chronic disorder of glucose metabolism, remains a health threat worldwide. To say the least this metabolic endocrine disorder is reported to have a devastating effect on male reproductive physiology. Ranging from a reduction in overall body weight, changes in reproductive organ weight; to its effect on male sex hormones, spermatogenesis, steroidogenesis as well as induction of oxidative stress observed in our study, the need to address both the cause and effect of diabetes would go a long way in addressing the menace of infertility.

The observed decrease in organ and body weight in diabetic rats could indicate altered metabolic process that could be linked to excessive tissue break down. [18,43]. However, the significant increase in the body weight and the lowering of blood glucose level to near normal following treatment with *L. micranthus* signify an improvement in the metabolic state and prevention of tissue damage associated with STZ-mediated hyperglycemic condition.

Several reports have shown that production of reactive oxygen species (ROS) is favored during chronic hyperglycemia leading to oxidative damage in a variety of tissues/organs [18, 44]. A very close link between ROS and reproductive system dysfunctions in diabetes has been reported [45,46]. CAT act in tandem to SOD during radical scavenging processes. These enzymes detoxify the superoxide anion, thus converting it into H_2_O_2_ and water [47]. The decreased activity of SOD in diabetic rats, could be due to glycation of the enzyme as a result of hyperglycemia. Rambir et al. 2013 [48] proposed that decrease in activities of SOD and CAT in organs during a diabetic state may be due to over-production of ROS in diabetic animals. Administration of hydro-metanolic extract of *L. micranthus* and glibenclamide showed a significant increase in SOD and CAT activity in testes. The phytochemicals present in *L. micranthus* may either be scavenging the STZ metabolites or may be reducing oxidative stress by lowering blood glucose levels. The seleno-GSH-Px, works together with GST in the metabolism of H_2_O_2_ and organic hydroperoxides to non-toxic products at the expense of GSH. Reduced activities of these endogenous antioxidant key players occasioned by diabetes may result from radical-induced inactivation and glycation [48] as observed in the current study. Administration of *L. micranthus* and glibenclamide significantly increased GSH-Px and GST activities. The decrease in GSH content in the testis of diabetic rats, and its normalization in *L. micranthus* and glibenclamide-treated animals, revealed the antioxidative potential of *L. micranthus*. In the current study, the level of LPO was increased in the testes of STZ-induced diabetic rats. Administration of *L. micranthus* and glibenclamide to diabetic rats decreased the level of LPO in testicular microenvironment. The obvious influence of *L. micranthus* in testes of diabetic rats via augmentation of antioxidant status and decreased lipid peroxidation has further strengthened the *in vivo* antioxidant properties of *L. micranthus* extract and its possible acceptance in folklore medicine for treatment of diabetes.

The two distinct segments of Glycolysis: an energy-consuming stage which produces FDP from glucose, and a second stage which solely involves energy production via the degradation of FDP to pyruvate [45], are very important in spermatogenesis. Although Sertoli cells (SCs) and germ cells express all of the enzymes of the glycolytic pathway, it is well known that germ cells predominantly use the lactate produced by SCs for energy production [46,47]. These data indicate that the key enzymes (Hexokinase, G-6-PD and LDH) involved in the glycolytic stages of spermatogenesis were reduced respectively, and this could insinuate impaired spermatogenesis and steroidogenesis, increased oxidative stress and germ cell apoptosis [48–53].

Bcl-2 belongs to a family of intracellular proteins that are involved in apoptosis by regulating activation of procaspases. Bcl-2 is an anti-apoptotic protein that acts partly by blocking the release of cytochrome C from mitochondria [54]. Reduced Bcl-2 expression in testicular cells of untreated diabetic rats in the current study in both immunoreactivity and western blot analyses suggest increased apoptotic processes. However, administration of extract inhibited apoptotic changes in diabetic rats as indicated by an increase in Bcl-2 expression.

Histology and histomorphometry in the present study confirm the deleterious effects of diabetes on the microstructure of the testes. Particularly, the results have shown that STZ-induced diabetes causes alterations in the testes, such as degeneration of seminiferous tubular contents (spermatogenic cells and spermatozoa), and loss or atrophy of Leydig cells in adjacent interstitial areas. Additionally, diabetes resulted in decreased seminiferous tubule diameter, and reduction in number of Leydig cells. These findings corroborate those of previous reports; STZ-induced diabetes resulted in reduced tubular diameter, and increased the percentage of tubular alterations such as vacuolations, missing spermatogenic generations, degenerated cells and appearance of multinucleated cells [55–56]. Additionally, Khaneshi et al. [57] also showed reduced tubular diameter in STZ-diabetic rats as well as reduced cellular contents of tubules, and atrophy of Leydig cells. However, in another study, no testicular structural defects were seen, though, reduction in luminal sperm density and seminiferous tubule diameter was observed [58]. In yet another study, alteration in interstitial tissues and reduction in total number of interstitial Leydig cells was observed, however, seminiferous tubules were shown to be mostly intact [59]. Interestingly, the present study showed appearance of calcification in necrosed seminiferous tubule in one animal. Calcifications of vas deferens and seminal vesicles have previously been linked to occurrence of diabetes [60–62]. Guvel et al. [63] showed testicular microlithiasis in one out of twenty-two diabetic subjects. Testicular microlithiasis is formation of calcium deposits in lumen of seminiferous tubules [64]. Treatment of diabetic rats with glibenclamide or 200 mg/kg extract improved STZ-diabetic induced testicular histological alterations. Results indicate that 200 mg/kg extract treatment showed better improvement than glibenclamide treatment. However, 100 mg/kg extract treatment did not show any improvement in testicular histological alterations.

## Conclusion

Taken together, the findings from the present investigation demonstrated that administration of *L. micranthus* leaves extract significantly assuaged hyperglycemia-mediated testicular oxidative damage in rats rendered diabetic by STZ treatment via multiple mechanisms including inhibition of lipid peroxidation, enhancement of the antioxidant system, decreased testicular apoptosis, maintenance of sperm quality and preservation of the hypothalamus–pituitary–gonadal axes. This study provides convincing scientific support for the traditional use of *L. micranthus* leaves in the treatment of diabetes and its associated complications like male infertility.
